# Atrial Flutter Ablation and Risk of Right Coronary Artery Injury

**DOI:** 10.14740/jocmr1986w

**Published:** 2015-02-09

**Authors:** Basel Al Aloul, Gardar Sigurdsson, Selcuk Adabag, Jian-Ming Li, Richard Dykoski, Venkatakrishna N. Tholakanahalli

**Affiliations:** a Cardiac and Vascular Consultants, The Villages, FL, USA; b Cardiovascular Disease Division, University of Minnesota, Minneapolis, MN, USA; c University of Iowa Hospital and Clinics, Iowa City, IA, USA; d Cardiology Section, Veterans Affairs Medical Center, Minneapolis, MN, USA

**Keywords:** Atrial flutter, Coronary artery, Cardiac arrhythmia, Myocardial infarction, Radiofrequency catheter ablation

## Abstract

Radiofrequency ablation (RFA) of atrial flutter (AFL) is a commonly performed procedure with low risk of complications. Several case reports and animal studies cautioned about the risk of right coronary artery (RCA) injury following AFL ablation. This risk is due to the anatomic proximity of the RCA to the cavo-tricuspid isthmus where ablation is performed. We present a case report that demonstrates postmortem evidence of RCA injury following RFA of AFL.

## Introduction

Radiofrequency ablation (RFA) of atrial flutter (AFL) is a very common procedure performed to achieve rhythm control with high success rates. In patients with typical AFL, the long-term success rate of cavo-tricuspid isthmus (CTI) ablation is more than 90% [1]. The North American Society for Pacing and Electrophysiology reported that the incidence of acute myocardial infarction (MI) after RFA for atrial arrhythmias is < 0.1% [2]. In light of the high efficacy and low risk of procedural complications, catheter ablation is recommended as a class I therapeutic option for patients with recurrent AFL and for patient with poorly tolerated chronic AFL [2]. Potential complications of AFL ablation include MI, atrio-ventricular block, ventricular tachycardia, tamponade, pericardial effusion, deep venous thrombosis, pulmonary embolization and stroke. Data regarding coronary artery injury are sparse. Prior human and animal studies cautioned about the potential risk of coronary artery injury [3-10]. We present a case report that demonstrates postmortem evidence of right coronary artery (RCA) injury following AFL ablation.

## Case Report

A 58-year-old male with severe lung disease due to end-stage chronic obstructive pulmonary disease (COPD) requiring tracheal intubation and ventilation underwent CTI ablation for a new diagnosis of AFL causing hemodynamic instability. In the electrophysiology lab his ECG showed AFL with 4:1 conduction. AFL cycle length was 180 ms, preventing entrainment maneuver. Under direct fluoroscopy guidance, a Bard DECA catheter was advanced to the coronary sinus. Subsequently, an EPT Blazer II 8 mm ablation catheter was advanced to the right atrium and positioned in the CTI area at 6 o’clock position from the left anterior oblique view where ablation was performed at a power of 35 W and a temperature of 45 °C. After initial series of RFAs guided by temperature and impedance, the tachycardia cycle length slowed down to 190 ms. Therefore, entrainment was then attempted with pacing cycle length of 180 ms at the CTI area from 6 - 7 o’clock positions. However, the atrium was not captured. Further RFAs were performed along the CTI employing maximum power up to 100 W and maximum temperature of 60 °C for a maximum of 60 s. Ablation was performed across the CTI between 6 and 8 o’clock positions from the left anterior oblique view without a change in the cycle length. Therefore, coronary sinus catheter was then pulled back to be positioned at the lateral right atrial wall. The atrial activation sequence showed a pattern consistent with typical AFL. Then the coronary sinus catheter was repositioned inside the coronary sinus and entrainment was successful from the proximal coronary sinus, with post pacing interval minus tachycardia cycle length of < 10 - 15 ms. These findings were very supportive again of typical isthmus dependent AFL. Therefore, ablation was performed medially, and just posterior and anterior to coronary sinus os resulting in successful termination of AFL and resumption of sinus rhythm. Insurance burns and differential pacing was performed. Post-ablation trans-isthmus conduction time was 105 ms pacing from proximal coronary sinus to the low lateral right atrium. During the entire procedure the average temperature did not exceed 60 °C. Moreover, the impedance did not rise and there was no steam pops. The patient tolerated the procedure well without immediate complications. There was no ECG evidence of ischemia during or after the ablation procedure and no echocardiogram was performed. Eight days after the procedure the patient remained ventilator dependent due to severe bilateral pneumonia and COPD exacerbation and died later from respiratory failure. An autopsy was performed.

**Figure 1:**
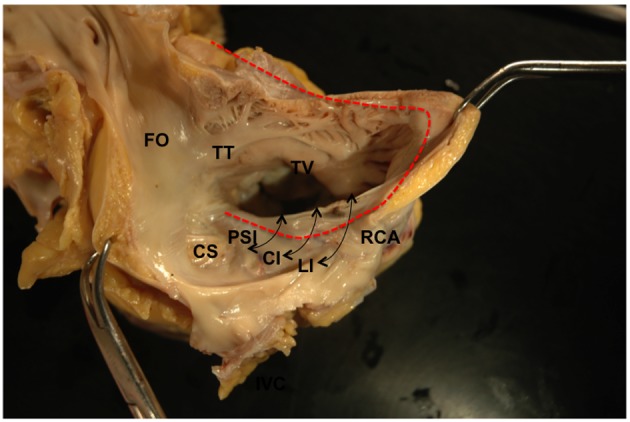
Gross examination of the right atrium shows white area of discoloration along the cavo-tricuspid isthmus region and an area of hemorrhage between the coronary sinus and the tricuspid valve. Three levels of the cavo-tricuspid isthmus are shown: lateral isthmus, central isthmus and paraseptal isthmus. A projected course of the right coronary artery (red line) demonstrates its anatomic relation to the cavo-tricuspid isthmus. CTI: cavo-tricuspid isthmus; FO: foramen ovale; TV: tricuspid valve; CS: coronary sinus; RCA: right coronary artery; TT: tendon of Todaro; LI: lateral isthmus; CI: central isthmus; PSI: paraseptal isthmus.

**Figure 2:**
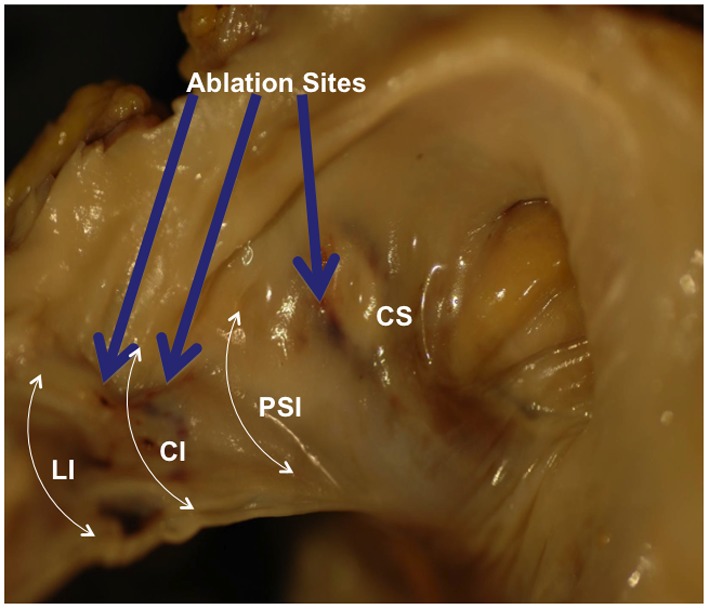
Magnified right anterior oblique view of the internal structures of the right atrium. Ablation sites are shown (blue arrows). LI: lateral isthmus; CI: central isthmus; PSI: paraseptal isthmus; CS: coronary sinus.

**Figure 3:**
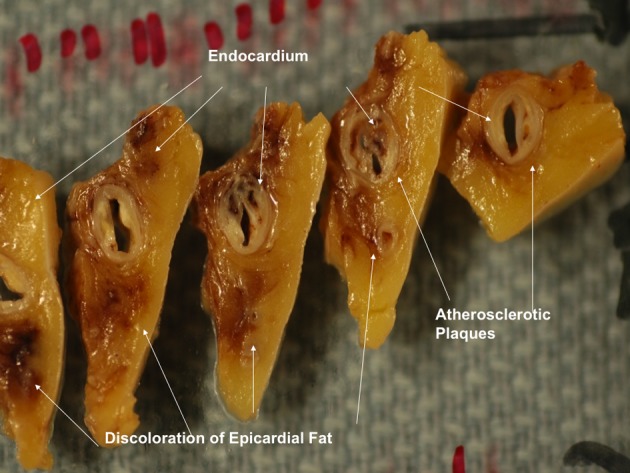
Magnified view of the distal right coronary artery shows brown discoloration of the epicardial fat.

**Figure 4:**
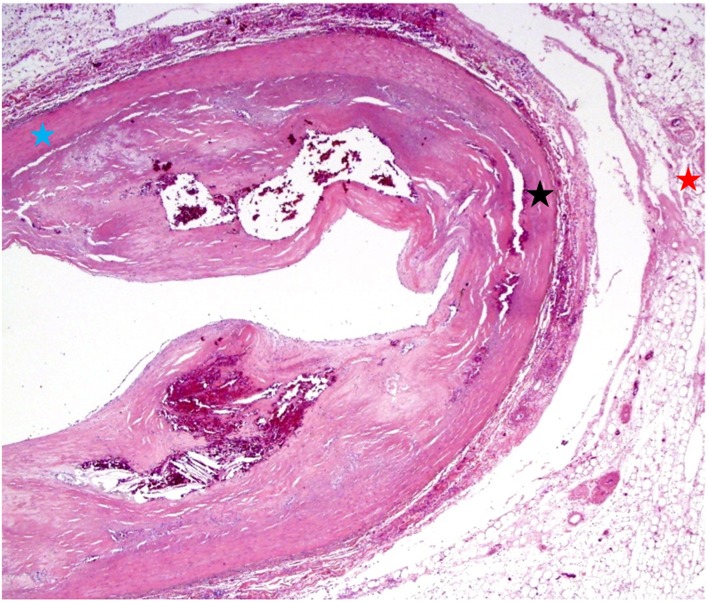
Microscopic examination shows cross-sections of the right coronary artery taken from the region of the ablation site demonstrating fat necrosis (red asterisk) and reactive changes to the fat and soft tissues of the right atrioventricular junction. Within the one-fifth of the vessel’s media, the smooth muscle cells have loss of nuclei (lack of blue colored nuclei) as shown by the black asterisk when compared to the normal smooth muscle cells with preserved nuclei (blue asterisk). Right coronary artery is narrowed by calcified atherosclerotic plaque with areas of acute plaque hemorrhage.

Gross examination of the right atrium showed a large area of hemorrhage and pale discoloration of the endocardium between the coronary sinus ostium, inferior vena cava (IVC) and the posterior annulus of tricuspid valve (Fig. 1). A closer view of the ablation site within the Eustachian ridge is shown on Figure 2. Examination of the distal RCA, within the right atrioventricular junction, just proximal to the posterior descending coronary artery (PDA), demonstrated an area of brownish discoloration of the epicardial fat measuring 40 mm in length and 15 mm in depth. The RCA within this segment was 80% narrowed by atherosclerotic plaque (Fig. 3). Microscopic examination of the RCA revealed necrosis and reactive changes to the surrounding fat and soft tissues. Within one-fifth of the vessel’s media, the smooth muscle cells had lost their nuclei (Fig. 4). Cross-sections of the RCA revealed lumen narrowing by calcified atherosclerotic plaque with areas of acute plaque hemorrhage. Microscopic examination of the right ventricle showed no evidence of infarction. However, left ventricular examination showed evidence of myocardial necrosis in the distal portion of the posterior wall surrounded by granulation tissue with hemosiderin-laden macrophages and lymphocytes.

## Discussion

Our case demonstrates that CTI RFA, a widely used treatment for AFL, may be responsible for coronary artery injury. Indeed our patient’s postmortem examination showed significant atherosclerosis but it also revealed RCA epicardial fat injury, acute plaque hemorrhage within the calcified atherosclerotic plaque and MI 8 days following RFA. Therefore, in this case we consider that AFL ablation may be responsible for the RCA injury. This complication is probably due to the anatomic proximity of the CTI to the RCA, the thin muscle layers between the atrial part of CTI and the RCA, the degree of power utilized for radiofrequency application and perhaps the size of catheter. It is not clear whether the clinical outcome was related to this complication. The severity of our patients’ lung disease probably played the biggest role in his outcome especially that his autopsy showed severe emphysema and diffuse pulmonary hemorrhage that made him difficult to ventilate.

The differential diagnosis of the pathological changes seen on autopsy can be due to any type of injury that can obstruct blood flow in the RCA during the ablation procedure including coronary spasm, acute coronary syndrome and thermal injury. However, since these changes are focally located in the epicardial fat in addition to myocardial necrosis in the distribution of the RCA, thermal injury from CTI RFA is the most likely culprit. RFA was performed guided by impedance and temperature monitoring. The power was gradually increased up to 100 W to achieve a maximum temperature of 60 °C. Higher power and temperature might have led to a greater insult. The rationale for use of higher power was because lower numbers did not work. Whether this is the main reason for the observed injury is unknown. Autopsy also, showed calcified atherosclerotic plaque with areas of acute plaque hemorrhage. The calcified atherosclerosis is most likely related to native coronary artery disease, but acute plaque hemorrhage can be due to either an acute coronary syndrome or direct thermal injury from the ablation catheter.

While there is general agreement that patients with AFL should be treated with RFA due to its high success rates and low risk of short-term complications, subclinical and long-term risk to the RCA is largely unknown. It has been shown that RFA in animals can directly damage coronary arteries and adjacent myocardium in eight out of 10 young pigs [3]. In this study, ablation in the right atrial aspect of the tricuspid valve annulus resulted in RCA injury at 48 h and at 6 months. Another study cautioned about the proximity of the coronary arteries to the common ablation sites of arrhythmias using coronary angiography [4]. On the other hand, a study of 150 patients undergoing catheter ablation of AFL reported a 2.7% procedure related complications but no coronary artery related complications [11]. We found four cases in the literature that reported RCA complications after AFL ablation. Ouali et al reported a patient who developed inferior ST segment elevation (STE) during RFA of AFL, which was treated with thrombolysis [5]. Moreover, Raio et al reported a patient who developed inferior STE immediately following AFL ablation, which was treated with percutaneous intervention [6]. Weiss et al reported a patient who died from severe heart failure and pneumonia 3 weeks after AFL ablation. Pathological analysis of the patient’s RCA showed intramural hemorrhage adjacent to the side of the ablation lesion but without involvement of the other layers [7]. Furthermore, Sassone et al reported a patient who died from cardiac rupture, as a complication of MI following RCA occlusion after AFL ablation [8].

RCA injury as a complication of RFA can be early or late. In all of previously mentioned reports including ours, RCA injury was early. However, there are reports of late coronary artery injury following RFA. Bertram et al reported two children with Epstein’s anomaly and Wolf Parkinson White syndrome, who developed late RCA stenosis after accessory pathway ablation. These two patients had normal coronary angiograms immediately after ablation but during subsequent evaluation for recurrent supra-ventricular tachycardia, they both had significant RCA stenosis [12]. Moreover, late occlusion of the left main coronary artery was reported in a young male 2 years after an uncomplicated, successful ablation of idiopathic left ventricular tachycardia [13]. Until now it is unclear whether AFL abaltion in particular can have long-term coronary complications. In general if a patient presents with RCA stenosis, we do not go back to check whether the patient has a prior history of AFL ablation. Measures to avoid this potential complication include, limiting power and temperature and monitoring impedance during RFA. Also, in selected cases use of intracardiac echo or angiography can be useful to identify ridges, pouches, catheter contact, and development of perforation or steam pops. For successful CTI ablation, it is important to have a stable temperature, while higher temperatures may cause tissue vaporization (i.e., steam pops) or charring, or formation of blood coagulum on the ablation electrode resulting in a rise in impedance that limits energy delivery and lesion formation which may lead to complications such as cardiac perforation or embolization [14].

In conclusion RCA injury may be caused by AFL RFA. In patients without chest pain or ECG changes following AFL ablation, the significance of this injury is unknown.

## References

[1] Willems S, Weiss C, Ventura R, Ruppel R, Risius T, Hoffmann M, Meinertz T (2000). Catheter ablation of atrial flutter guided by electroanatomic mapping (CARTO): a randomized comparison to the conventional approach. J Cardiovasc Electrophysiol.

[2] Blomstrom-Lundqvist C, Scheinman MM, Aliot EM, Alpert JS, Calkins H, Camm AJ, Campbell WB (2003). ACC/AHA/ESC guidelines for the management of patients with supraventricular arrhythmias--executive summary. a report of the American college of cardiology/American heart association task force on practice guidelines and the European society of cardiology committee for practice guidelines (writing committee to develop guidelines for the management of patients with supraventricular arrhythmias) developed in collaboration with NASPE-Heart Rhythm Society. J Am Coll Cardiol.

[3] Paul T, Bokenkamp R, Mahnert B, Trappe HJ (1997). Coronary artery involvement early and late after radiofrequency current application in young pigs. Am Heart J.

[4] Hasdemir C, Yavuzgil O, Payzin S, Aydin M, Ulucan C, Kayikcioglu M, Can LH (2007). Angiographic analysis of the anatomic relation of coronary arteries to mitral and tricuspid annulus and implications for radiofrequency ablation. Am J Cardiol.

[5] Ouali S, Anselme F, Savoure A, Cribier A (2002). Acute coronary occlusion during radiofrequency catheter ablation of typical atrial flutter. J Cardiovasc Electrophysiol.

[6] Raio N, Cohen TJ, Daggubati R, Marzo K (2005). Acute right coronary artery occlusion following radiofrequency catheter ablation of atrial flutter. J Invasive Cardiol.

[7] Weiss C, Becker J, Hoffmann M, Willems S (2002). Can radiofrequency current isthmus ablation damage the right coronary artery? Histopathological findings following the use of a long (8 mm) tip electrode. Pacing Clin Electrophysiol.

[8] Sassone B, Leone O, Martinelli GN, Di Pasquale G (2004). Acute myocardial infarction after radiofrequency catheter ablation of typical atrial flutter: histopathological findings and etiopathogenetic hypothesis. Ital Heart J.

[9] Solomon AJ, Tracy CM, Swartz JF, Reagan KM, Karasik PE, Fletcher RD (1993). Effect on coronary artery anatomy of radiofrequency catheter ablation of atrial insertion sites of accessory pathways. J Am Coll Cardiol.

[10] Bokenkamp R, Wibbelt G, Sturm M, Windhagen-Mahnert B, Bertram H, Hausdorf G, Paul T (2000). Effects of intracardiac radiofrequency current application on coronary artery vessels in young pigs. J Cardiovasc Electrophysiol.

[11] Calkins H, Canby R, Weiss R, Taylor G, Wells P, Chinitz L, Milstein S (2004). Results of catheter ablation of typical atrial flutter. Am J Cardiol.

[12] Bertram H, Bokenkamp R, Peuster M, Hausdorf G, Paul T (2001). Coronary artery stenosis after radiofrequency catheter ablation of accessory atrioventricular pathways in children with Ebstein's malformation. Circulation.

[13] Pons M, Beck L, Leclercq F, Ferriere M, Albat B, Davy JM (1997). Chronic left main coronary artery occlusion: a complication of radiofrequency ablation of idiopathic left ventricular tachycardia. Pacing Clin Electrophysiol.

[14] Feld GK, Birgersdotter-Green U, Narayan S, Wilber DJ, Packer DL, Stevenson WG (2008). Diagnosis and ablation of typical and reverse typical (type 1) atrial flutter. Catheter ablation of cardiac arrhythmias.

